# Supplementary material for: CLINICAL DIAGNOSIS CODES IDENTIFY PATIENTS UNLIKELY TO RECEIVE ORDERS FOR FECAL IMMUNOCHEMICAL TESTS
Source: medRxiv. 2025 Jun 9:2025.06.07.25327375. Preprint. [Version 1] doi: 10.1101/2025.06.07.25327375 (PMC12204409; doi:10.1101/2025.06.07.25327375)

medRxiv preprint doi: https://doi.org/10.1101/2025.06.07.25327375; this version posted June 9, 2025. The copyright holder for this preprint (which was not certified by peer review) is the author/funder, who has granted medRxiv a license to display the preprint in perpetuity.
It is made available under a CC-BY 4.0 International license .

Supplementary Figure. 1. Count and proportion of patients in the cohort with FIT ordered according to Charlson Comorbidity Index (CCI).

Supplementary Figure 2. ICD-10 codes (n = 1,2*15*) distributed according to their odds ratio and patient count, plotted on a logarithmic scale. Codes with an odds ratio less than 0.6 are represented by the vertical red dashed line. Codes marked with an “x” *were not significant* (n = *1112*), codes with hollow circles were significant *and OR > 0.6* (n = *41*), and solid circles were significant *and OR* < *0.6* (n = 6*2*). *Significance was determined to be* P < *0.05* based on two-sided Fisher’s exact tests. Benjamini−Hochberg correction for multiple tests was applied. Rank is the ratio of patient count to odds ratio, such that high-ranked codes have a low odds ratio and high patient count.

medRxiv preprint doi: https://doi.org/10.1101/2025.06.07.25327375; this version posted June 9, 2025. The copyright holder for this preprint (which was not certified by peer review) is the author/funder, who has granted medRxiv a license to display the preprint in perpetuity.
It is made available under a CC-BY 4.0 International license .

Supplementary Figure 1. Count and proportion of patients in the cohort with FIT ordered according to Charlson Comorbidity Index (CCI).

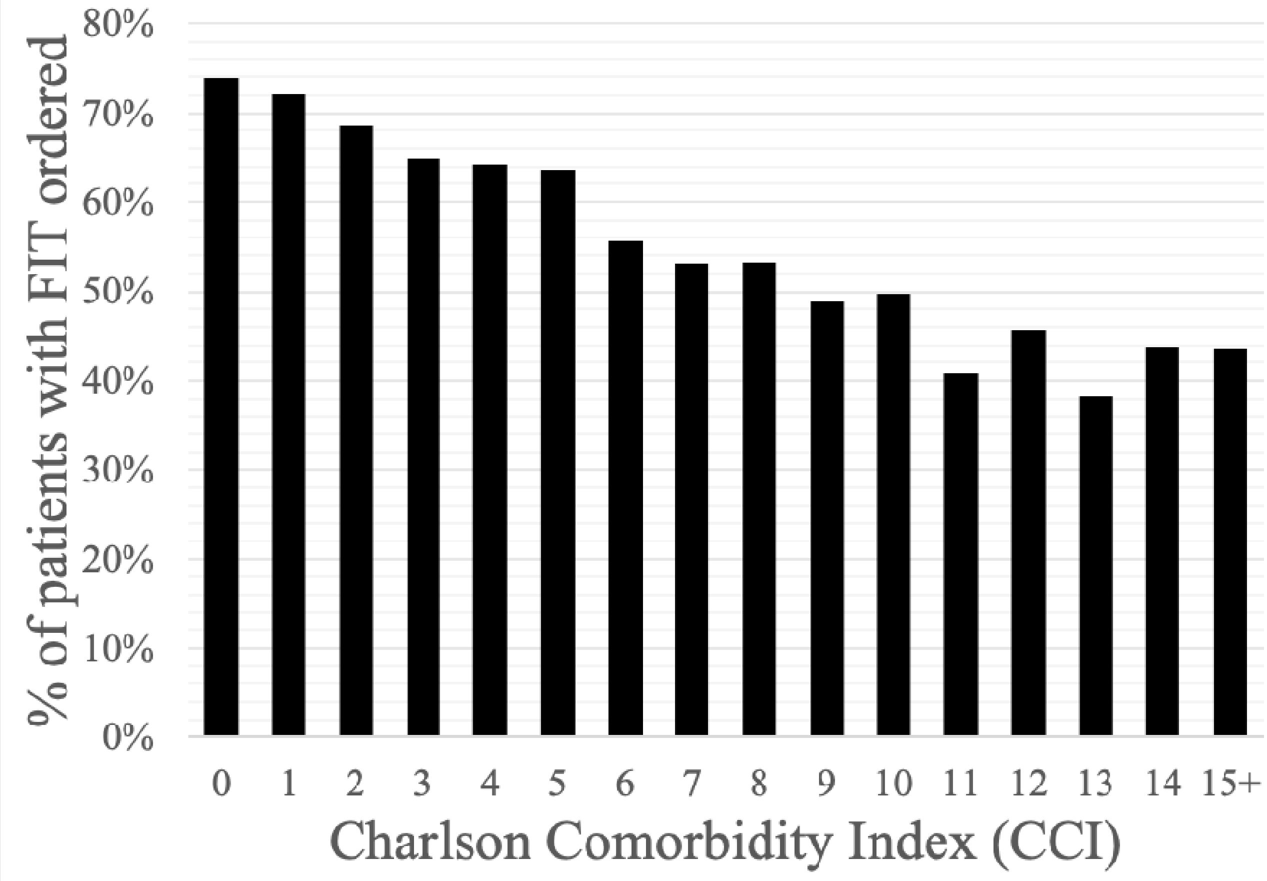

medRxiv preprint doi: https://doi.org/10.1101/2025.06.07.25327375; this version posted June 9, 2025. The copyright holder for this preprint (which was not certified by peer review) is the author/funder, who has granted medRxiv a license to display the preprint in perpetuity.
It is made available under a CC-BY 4.0 International license .

Supplementary Figure 2. ICD-10 codes (n = 1,215) distributed according to their odds ratio and patient count, plotted on a logarithmic scale. Codes with an odds ratio less than 0.6 are represented by the vertical red dashed line. Codes marked with an “x” were not significant (n = 1,112), codes with hollow circles were significant and OR > 0.6 (n = 41), and solid circles were significant and OR < 0.6 (n = 62). Rank is the ratio of patient count to odds ratio, such that high-ranked codes have a low odds ratio and high patient count.

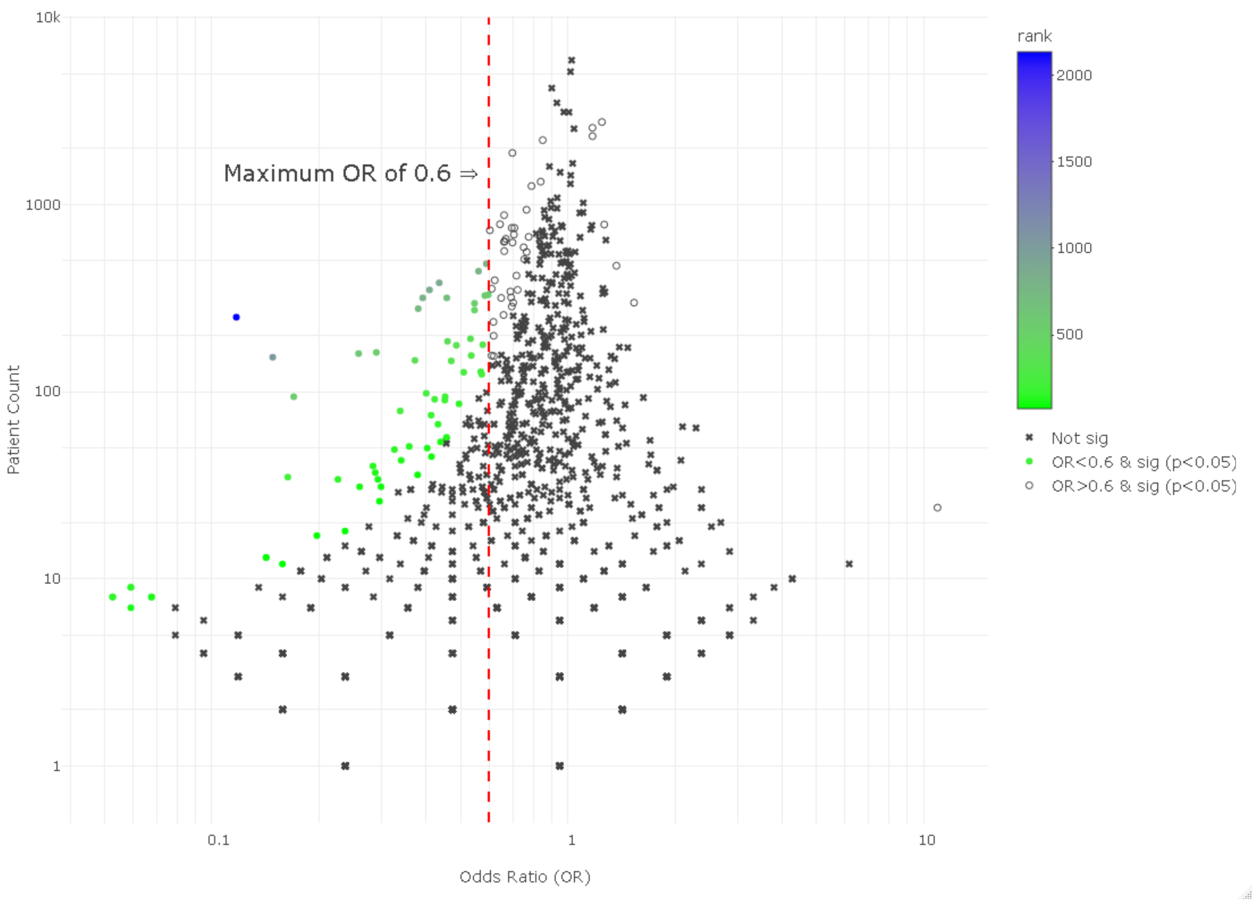

Supplement: Supplement 2 [file NIHPP2025.06.07.25327375v1-supplement-2.pdf]
